# Individual and Public-Program Adaptation: Coping with Heat Waves in Five Cities in Canada 

**DOI:** 10.3390/ijerph8124679

**Published:** 2011-12-16

**Authors:** Anna Alberini, Will Gans, Mustapha Alhassan

**Affiliations:** 1 AREC, 2200 Symons Hall, University of Maryland, College Park, MD 20742, USA; Email: wgans@arec.umd.edu; 2 School of Biological Sciences and Institute for a Sustainable World, Queen’s University Belfast, Medical Biology Centre, 97 Lisburn Road, Belfast BT9 7BL, UK; 3 Department of Agricultural and Resource Economics, Clark B-320, Colorado State University, Fort Collins, CO 80523, USA; Email: malhassa@rams.colostate.edu

**Keywords:** Heat Alert Response Systems (HARS), Heat/Health Watch/Warning Systems (HHWW), excessive heat, heat-related illness

## Abstract

Heat Alert and Response Systems (HARS) are currently undergoing testing and implementation in Canada. These programs seek to reduce the adverse health effects of heat waves on human health by issuing weather forecasts and warnings, informing individuals about possible protections from excessive heat, and providing such protections to vulnerable subpopulations and individuals at risk. For these programs to be designed effectively, it is important to know how individuals perceive the heat, what their experience with heat-related illness is, how they protect themselves from excessive heat, and how they acquire information about such protections. In September 2010, we conducted a survey of households in 5 cities in Canada to study these issues. At the time of the survey, these cities had not implemented heat outreach and response systems. The study results indicate that individuals’ recollections of recent heat wave events were generally accurate. About 21% of the sample reported feeling unwell during the most recent heat spell, but these illnesses were generally minor. Only in 25 cases out of 243, these illnesses were confirmed or diagnosed by a health care professional. The rate at which our respondents reported heat-related illnesses was higher among those with cardiovascular and respiratory illnesses, was higher among younger respondents and bore no relationship with the availability of air conditioning at home. Most of the respondents indicated that they would not dismiss themselves as “not at risk” and that they would cope with excessive heat by staying in air conditioned environments and keeping well hydrated. Despite the absence of heat outreach and education programs in their city, our respondents at least a rough idea of how to take care of themselves. The presence of air conditioning and knowledge of cooling centers is location-specific, which provides opportunities for targeting HARS interventions.

## 1. Introduction

The 2001 and 2007 Intergovernmental Panel on Climate Change (IPPC) reports [1,2] warn that an increase in the frequency and/or intensity of heat waves will raise heat-related premature mortality, primarily among the elderly and the urban poor, with the largest increases in thermal stresses occurring in cities in temperate regions. This has resulted in adoption or at least consideration of public programs that help curb the mortality and morbidity effects of extremely hot weather.

Epidemiological investigations based on the statistical analysis of death counts and death certificates have found that mortality increases—sometimes very sharply—above the long-term average during heat waves. Such excess mortality is about an order of magnitude larger than directly observed heat mortality [3] (≈1800 and 180 deaths per summer in the US, respectively; US EPA [4]). 

Most premature deaths are attributed to cardio- and peripheral vascular, cerebrovascular, and respiratory causes [5,6,7,8,9,10,11,12,13,14,15]. The adverse health effects of heat waves are compounded by the poor air quality that sometimes accompanies them [9]. Individuals at risk during heat waves include infants, the elderly, those with existing cardio-, cerebrovascular and respiratory conditions, and individuals on certain medications. Protections include staying in climate-controlled environments [11,16,17], behavioral changes, and community-wide planning and warning systems. 

Compared to other countries, in Canada there have been few quantitative assessments of the adverse health effects of heat waves [18]. Extreme heat events are estimated to cause an average of 120 deaths in the City of Toronto alone per year [19], and it is predicted that heat-related premature deaths among the elderly could reach 144–447 per year in the Toronto-Niagara Region by the mid–2020s [20]. 

Concern about these effects has prompted the Canadian government to initiate campaigns to educate the public about the health risks of excessive heat, warning systems and emergency response programs. For these interventions to be effective, it is important to understand how people seek and receive information about extreme heat, if they have any experience with heat-related illnesses, and if they have access to protections from excessive heat (including air conditioning and cooling centers). This information can be collected through surveys of the population. 

Previous survey work about this topic has, however, been limited. Sheridan [21] interviewed elderly persons in Toronto, Canada, to assess awareness of excessive heat alerts and behavioral changes associated with the alert. Kalkstein and Sheridan [22] interviewed a convenience sample (persons at a shopping mall in Phoenix) to document their knowledge of the heat advisory and their behavior, but older individuals and other subjects at risk are underrepresented in their sample. Both Toronto and Phoenix have well-established heat response systems, making it difficult to extrapolate the results to cities that are yet to set up such systems. 

What is reported here is a study in five such cities. In early September 2010, at the end of one of the hottest summers on record [23], we conducted a survey of the Canadian public to investigate the abovementioned issues. The survey was commissioned by the Policy Research Initiative of the Government of Canada and Health Canada, and was administered to residents of five metropolitan areas in Canada. Three of these metropolitan areas (Winnipeg, MB, Windsor, ON, and Fredericton, NB) had been selected for an outreach program about excessive heat risks and for a pilot heat Alert and Response System (HARS). The educational materials were prepared by Health Canada [24], but the individual city governments would be in charge of the actual implementation of the program [25]. 

We added two more metropolitan areas that are similar for climate, geography and population—Regina, SK, and Sarnia, ON—to Winnipeg and Windsor, respectively, but had no plans for public outreach or HARS programs. At the time of this survey, no program had been implemented in any of these five cities, and so the purpose of our research was to gather information about pre-program knowledge of heat-related health risks and ways of coping with the heat. (In the future, we hope to be able to compare pre- and post-program knowledge using additional rounds of surveys that will take advantage of this “control” *v.* “treatment” design.) 

We note that at least two of the cities covered in this study (Windsor and Sarnia) lie in what Bassil *et al.* [26] consider “heat wave zones” (e.g., Ontario). These authors note that summers are generally cooler in Canada than in the US, including in the heat wave zones, and that, due to lower exposure to hot summer conditions, Canadians may be less physiologically adapted to heat episodes when they do occur.

Briefly, we find that individuals’ recollections of recent heat wave events were generally accurate. Our Fredericton, NB, sample was perhaps the group that was the most attuned to registering excessive heat, given the generally cool summers in the Atlantic Provinces of Canada. About 21% of the sample reported feeling unwell during the most recent heat spell, but these illnesses were generally minor. Only in 25 cases out of 243 were these illnesses confirmed or diagnosed by a health care professional. As expected, the rate at which our respondents reported heat-related illnesses (whether or not diagnosed by a health care professional) was higher among those with cardiovascular and respiratory illnesses. 

We were surprised that the younger individuals in our sample were, all else the same, more likely to report heat-related illnesses. We conjecture that this may be the result of spending more time outdoors, confusing air pollution-related symptoms with heat-related illnesses, or misclassification of symptoms. Even more surprising, having air conditioning at home was not related to the likelihood of reporting symptoms.

Most of the respondents indicated that would adopt common-sense, and medically appropriate, ways of coping with the heat (such as staying in air conditioned environments and keeping well hydrated), if a heat wave started tomorrow, and were especially proactive with children and elderly persons they are in charge of. In general, our respondents’ awareness of measures to protect themselves from the heat suggest is consistent with the figures reported in [21] for an elderly sample in Toronto, a city that has a well-established heat response system and where in the Summer of 2010 alone there were 5 days with heat alerts (three in May, one in July and one in August) and 11 days with extreme heat alerts (three in May, four in July, two in August and two in early September; see [27]). 

The remainder of this paper is organized as follows. Section 2 describes heat alert and response systems in Canada. Section 3 describes the survey questionnaire. Section 4 describes the sampling frame and provides descriptive statistics of the data. In Section 5 we examine the respondents’ experience with excessive heat. In Section 6 we study heat-related illness and in Section 7 air conditioning and its mitigation effects. In Section 8 we study how people would respond to alerts about an imminent heat wave, and in Section 9 their knowledge of cooling centers and perceptions of individuals at risk. Section 10 concludes. 

## 2. Heat Response Systems

Physiological adaptation takes place in the presence of excessive heat, and individuals become acclimated in ways that depend on age, gender, health status and cardiovascular fitness levels [28]. In addition, individuals can protect themselves from excessive heat by simply by spending more time in climate-controlled environments, using appropriate clothing, drinking more water, and refraining from strenuous exercise at the hottest hours of the day. 

At many locales, adaptation to excessive heat is further enhanced by government programs. Heat/Health Warning Systems (HHWSs) are considered a promising public health tool to reduce the adverse impacts of excessive heat on human health. Briefly, they consist of (i) preparations before the onset of excessive heat; (ii) meteorology-based warning systems; (iii) rapid and coordinated actions during heat waves; (iv) criteria and procedures for deactivating the plan, and (v) evaluations following the response activities and outcomes [29,30].

In the US, the National Weather Service (NWS) has been issuing excessive heat advisories, watches and warnings since 1993 [31,32]. The NWS alerts the population and issues advice on how people can protect themselves and others from the adverse health effects of heat waves, but the responsibility of actually setting up and running public health protection programs usually falls on individual cities and counties. Specific measures include opening and operating cooling centers, extending public swimming pool hours, distributing fans or air conditioners, offering nursing and medical advice over the phone, having medical staff or volunteers visit susceptible individuals (the elderly and those with mobility impairments), and others. Cities and states can also order the utilities to refrain from suspending service for non-payment, so that the poor can use fans and air conditioners without interruptions in the electricity service, and can distribute federal funding to help pay for electricity bills. 

City heat response program may issue alerts to the population based on the NWS heat advisories, watches and warning, or on alternate criteria more closely linked to the vulnerability of the local population. Systems of this kind are in place in many Midwestern and eastern US cities, and Ebi *et al.* [33] estimate that the Philadelphia HHWS avoided 117 heat-related fatalities over the first three years since its inception. 

In Canada, HHWWs are termed Heat Alert and Response Systems (HARSs). The Heat Advice Program in Canada [34] provides information about health risks, ways of reducing them (via cooling, clothing, behavioral changes, *etc*.) and identifies individuals at risk.

Clean Air Partnership [20] reviews existing programs in Canada, reporting that as of summer 2007, there were only two major metropolitan areas that had a working heat response program: The City of Toronto, along with many communities in the Greater Toronto Area (GTA), and Montreal. These two programs are also described in [35]. 

The various cities and communities within these metro areas adopted a variety of triggers for issuing advisories or warnings. These criteria include (i) spatial synoptic criteria; (ii) a humidex threshold; and (iii) minimum and maximum temperature thresholds. 

In 2000 the City of Toronto adopted a spatial synoptic classification system developed by Larry Kalkstein and Scott Sheridan at the University of Delaware. This method is location‐specific and works by identifying air masses or weather types historically associated with increases in mortality at a given locale. Unlike other watch-warning systems, this method takes into account the negative impact of several consecutive days of oppressive weather, as well as the fact that heat waves earlier in the year are more dangerous than those in late summer. Alerts are issued when oppressive weather is forecast and the likelihood of excess mortality is determined to exceed 65% (Heat Alert) or 90% (Extreme Heat Alert). Because it is based on the likelihood of excess mortality, as opposed to predicted excess mortality counts, the Toronto system is different from similar systems developed by Kalkstein and Sheridan for several US cities. The Peel region in the GTA adopted a spatial synoptic system in 2006. 

The Kalkstein-Sheridan system has advantages and disadvantages. On the one hand, it caters to local conditions and to the characteristics of the local population. On the other hand, it is a complex system that relies on a large amount of data and is not easily calculated or verified by third parties. There are concerns that alerts and warnings are called too often. There are also challenges with implementing the system in smaller jurisdictions: The smallest metropolitan area for which a synoptic classification system has been developed is approximately 500,000 people. 

Other communities in the GTA rely on humidex thresholds, a measure that combines heat and humidity to reflect perceived temperature. Alerts are typically issued when the maximum daily humidex value is expected to exceed 40 °C, or when humidex values are expected to exceed 36 °C, for an extended period of time (3 days). Potential limitations of humidex-based triggers include that this system does not account for variability in human responses to heat, acclimatization over the summer months or the effects of several consecutive days of high heat and warm nights. Heat thresholds based on the humidex index are not targeted to specific populations but assume that temperature affects all people equally, regardless of the time of year, geographic location or heat wave duration.

In Montreal, a heat alert is triggered by a forecast of three consecutive days with lows of 20 °C or greater and highs of 33 °C or greater. Thresholds are geographically specific and take into account regional characteristics. 

In sum, hot weather response plans vary in scope, detail and precision, but typically consist of one or more of the following components:

- Procedures for alerting municipal staff, community agencies and the public to the occurrence of extreme heat;
- Procedures to communicate to the public and organizations that work with at-risk groups the health risks associated with extreme heat and heat‐safety information; and
- Procedures for rolling out public health intervention activities that typically include, but are not limited to, opening cooling centers and extending the operating hours of municipal swimming pools and other facilities.

The Clear Air Partnership document [20] emphasizes the inconsistencies among criteria and heat emergency responses across different communities, including the fact that the responsibility for issuing warnings vary with government tier by location. Staff in charge of heat wave programs in the GTA area and in Montreal typically bemoaned the lack of funding, the difficulty of identifying populations at risk, and other operational difficulties. Since at several locales heat wave responses are folded within air pollution alerts (heat waves are often, but not always, accompanied by poor air quality episodes), they also mentioned incorrect behavioral responses due to confusing and possibly conflicting alert messages. For example, some people refused to use air conditioning during a heat wave, in the mistaken belief that this would aggravate exposure to air pollution. 

This latter point underscores that the effect of excessive heat outreach programs and alerts presumably depends on the knowledge that people already have about risks and precautions. 

Earlier research has also examined and proposed explanations for how people process hazardous weather risk communication and alerts, including decision heuristics that undermine the effectiveness of emergency warnings. A recent investigation in Canada is Silver and Conrad [36], who study perceptions of and behavioral changes in response to severe weather events, which include thunderstorms, tornadoes, freezing rain, heavy rain, dust storms, blizzards, heavy snowfalls, frost, fog and wind chill. Heat waves are not covered in their study. They administer in-person surveys in Nova Scotia, at both urban and rural locations, reporting that the frequency, mode and provider with which individuals check the weather forecast, and the confidence in the accuracy of the forecast, tend to be age-specific. The ability to change behavior was related to one’s more or less flexible work schedule. 

Because much of this research is focused on weather hazards that imply losses to property and “accidental” deaths due to floodwaters, collapsing buildings, fires, *etc.*, it is unclear if its findings apply to heat wave situations. Kalkstein and Sheridan [22] conducted surveys of individuals intercepted at shopping malls in Phoenix, reporting that awareness of the heat advisory in place at the time was universal. The sample, however, underrepresented the elderly and persons at risk, and so it is difficult to extrapolate the findings to the general population. 

Sheridan [21] conducted telephone interviews of individuals in three US cities and one Canadian city (Toronto). While knowledge of the heat warning system was virtually universal, few survey participants changed behavior: They did not consider themselves vulnerable and did not think that the mitigation plan messages applied to them. The survey also revealed confusion between air quality and heat wave precautions. 

## 3. Questionnaire Description

Our survey questionnaire is comprised of five sections and is reported in the supplementary. In Section 1, we ask the respondent to recall the heat spells of summer 2010 (or previous summers) and to describe the most recent one for us. How did the respondent feel? Was it hot and dry? Hot and humid? With no wind? Were the nights too warm?

Next, we ask the respondent if he or she experienced any adverse health effects during this heat event, and how severe this heat-related illness was. We further inquire whether it was diagnosed by a health care professional, and whether it interfered with normal daily activities (or worse, required seeing a doctor or going to a medical facility). 

For policy purposes, it is important to establish whether people heed announcements and the weather forecast in advance of heat wave events, so we ask respondents if they knew ahead of time that it would be very hot, and how they found out. 

We investigate individual opportunities for coping with the heat by asking respondents what they would do if a three-day heat wave started tomorrow. In three separate questions, we ask people what they would do to take care of themselves, their small children, and elderly family members, relatives, friends or neighbors. Response options include staying inside, using various cooling devices, drinking plenty of water, going to a swimming pool or other body of water, going to the movies or the shopping mall because these places are usually air conditioned, and others. We also inquire if the respondent has heard of cooling centers in his city or elsewhere, and has ever used one, and how he would find out if one or more exist in his city. 

Section 2 of the questionnaire is about the respondent’s attitude towards (i) various environmental, economic and social problems; (ii) government and individual responsibilities during extreme weather events, including heat waves; and (iii) perceptions of extreme weather, natural disasters, and other risks (such as traffic accidents, terrorism, air pollution and forest fires). 

Section 3 inquires about features of the neighborhood where the respondent lives and the respondent’s home that might affect exposure to excessive heat or ways in which he or she copes with the heat. Specifically, we ask the respondent if he or she lives in an urban, suburban or rural environment, what type of home he lives in, and whether air conditioning is available in the home or at least in the common areas of a multi-family housing building. The presence of trees, grass and open spaces in the neighborhood can affect the temperature in that neighborhood and is considered an important determinant of the so-called “urban heat island effect,” so we inquire whether there are trees in the immediate vicinity of the respondent’s home. 

Section 4 queries the respondent’s about his or her health status, smoking habits, and reliance on assistance services for the elderly and mobility impaired. Section 5 contains the usual sociodemographics (age, gender, family status, income, employment, and education). 

## 4. Survey Administration, Sampling Plan and Sample

The questionnaire was self-administered on-line by respondents in five metropolitan areas in Canada (see Table 1). These cities were selected to ensure good geographical coverage of eastern and western Canada, comprise both urban and rural areas, and contain populations that are likely to be vulnerable to excessive heat (e.g., the elderly). Even more important, at the time they did not have a HHWW program in place, but three of them (Winnipeg, Windsor, and Fredericton) had been selected as the site of a government outreach and education program about excessive heat at a later date. We added two more cities—Regina and Sarnia—that are similar to Winnipeg and Windsor for climate and population, but had no HHWW and no plans for an outreach program. 

**Table 1:** Study locations and sample sizes. N = 1141.

Locations selected to receive the outreach program		Control locations	
Location	N (percent of the sample)	Location	N (percent of the sample)
Fredericton, NB	112 (9.82)	n/a	-
Winnipeg, MB	501 (43.91)	Regina, SK	173 (15.16)
Windsor, ON	238 (20.86)	Sarnia, ON	117 (10.25)

Respondents were recruited from the IPSOS i-say panel, and the sample was to follow quotas for city, gender and age in proportion to the population shares. The survey took place on 2–18 September 2010, with the majority of the questionnaires being completed within the first ten days of that month. We received a total of 1141 completed questionnaires. Women accounted for 55% of the sample, and the distribution of the respondents by age was similar across cities. Overall, 18.5% of the respondents were aged 25–34, 45% were aged 35–54, 16% were aged 55–64, 15% were aged 65–74, and 5.5% were 75 and older.

About one-third of the sample has a college degree or equivalent education, or has done graduate work. About 57% work full- or part-time, 3.77% are looking for work, and students account for less than 4% of the sample. Homemakers and retired persons account for 6% and 28.57% of the sample, respectively. The median household income is between CAN$60,000 and CAN$70,000 (2010 CAN$).

Recent epidemiological research emphasizes the importance of good cardiovascular fitness during heat waves. For this reason, we elicited information about the respondent’s health status. The respondents described themselves as being in good (34.88%), very good (37.42%) or even excellent health (14.27%) compared to others the same age. Only 14% of the respondents said that they were in fair or poor health.

More details about the respondent’s cardiovascular and respiratory health are displayed in Table 2. High blood pressure and high levels of low-density cholesterol are common complaints (almost 30% and 27%, respectively) and 13% of the respondent report having asthma. Moreover, about 11.57% of the respondents have had to go the hospital or the emergency room in the last 5 years because of cardiovascular or respiratory illnesses, or cancer. 

**Table 2:** Health conditions reported by the respondents.

Description	Percent
Diabetes	11.04
High Blood Pressure	29.54
High Levels of LDL Cholesterol	26.91
Coronary disease	4.38
Angina	3.77
myocardial infarction	3.33
Stroke	2.02
any other cardiovascular disease	2.72
Emphysema	1.14
Chronic bronchitis	4.21
Asthma	13.06
Other respiratory problems	7.10
Cancer	5.17

When attention is restricted to the elderly persons that the respondent takes care of, 44.17% of these persons have a chronic cardiovascular or respiratory condition. A total of about 64% of them have some form of mobility impairment (minor for 26% of them, moderate for some 24%, and major for 13.6%). 

Very few of the respondents (a total of 26) received regular deliveries of meals, groceries, and medicines at home. Six received regular doctor visits at home, and no one reported living in nursing homes with assisted living.

## 5. Experience with Excessive Heat

Over 95% of the respondents had spent the summer of 2010 in their normal area of residence (with occasional out-of-town trips, reported by 40% of the respondents). 

**Figure 1:**
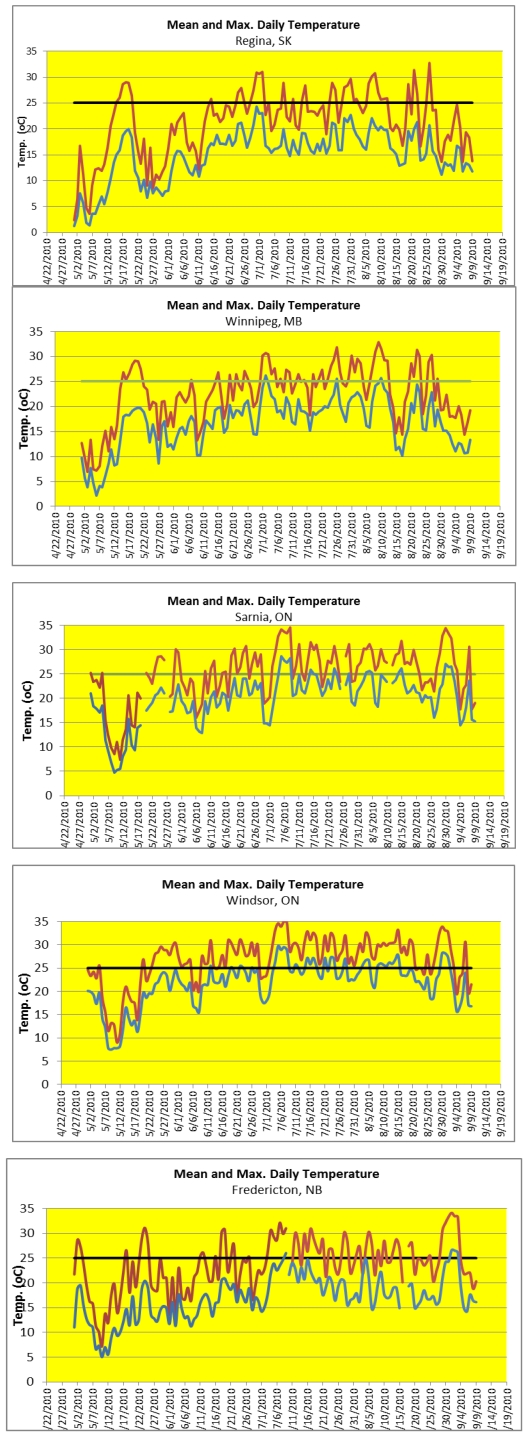
Daily average and maximum temperature by city. Summer 2010.

Graphs of May–September 2010 daily average and maximum temperatures in each city are displayed in Figure 1. The graphs show that there were unseasonably hot days in the second half to end of May. Temperature kept rising over June, and July and most of August were consistently hot, especially in Ontario and New Brunswick. The last few days of August and early September—just before or during the survey—were very hot. Table 3 displays the number of “hot” days by city, where a “hot” day is defined as one where the maximum temperature exceeds a specified threshold, confirming that Ontario and New Brunswick experienced the largest number of hot days.

Table 4 reports the number of heat “spells,” where a spell is an episode consisting of a specified number of consecutive days with maximum temperature greater than or equal to 30 °C. The table shows that Windsor and Sarnia had the most numerous episodes, but that the longest hot episode (six consecutive days with maximum temperature above 30 °C) occurred in Fredericton at the beginning of September—just when our respondents were participating in the survey. 

**Table 3:** Number of hot days by city between 1 May and 10 September 2010.

	Number of days with max temp > …			
	22 °C	25 °C	27 °C	30 °C
Regina	70	39	23	5
Winnipeg	79	50	47	11
Sarnia	98	76	52	25
Windsor	113	96	75	39
Fredericton	83	54	37	15

**Table 4:** Number of episodes with 2 or more consecutive days with maximum temperature above 30 °C between 1 May and 10 September 2010 by city.

	2 consecutive days	3 consecutive days	4 consecutive days	5 consecutive days	6 consecutive days
Regina	1	1	0	0	0
Winnipeg	1	1	0	0	0
Sarnia	0	2	1	1	0
Windsor	5	0	2	2	0
Fredericton	2	0	0	0	1

When we asked them whether there were times in the summer of 2010 (defined as 1 June to 15 September 2010) when the weather felt extremely hot, 84.27% of the respondents told us that this was the case. The number of such heat episodes (where an “episode” was two or more days) ranged from 1 to 100, for an average of 7.45. The median was 4 episodes. 

The survey questionnaire asked respondents to pinpoint the periods between 1 June and 15 September 2010, when they experienced extremely hot weather, and, as shown in Table 5, July and August were the hottest months, especially the second half of July and the first half of August. 

**Table 5:** Respondent-reported heat episodes in summer 2010.

Period	Number who selected this option	Percent
1–15 June	76	8.30
16–30 June	182	19.87
1–15 July	369	40.28
16–31 July	510	55.68
1–15 August	468	51.09
16–31 August	391	42.69
1–15 September	112	12.23
Don’t remember	160	17.47

As shown in Figure 1 and in Table 5, respondents in Ontario and New Brunswick were experiencing a heat wave at the time they took the survey (early September). By that time, the weather had cooled off in Manitoba and Saskatchewan. As shown in Table 6, the respondents’ recollections generally mirrored these records quite accurately. New Brunswick respondents, who are presumably unaccustomed to hot summers, were acutely aware of the hot spell in late August/early September. 

**Table 6:** Respondent-reported heat periods by area of residence. Percentages of column totals.

	Regina, SK	Winnipeg, MB	Sarnia, ON	Windsor, ON	Fredericton, NB
1–15 June	3.30	5.01	10.28	14.86	9.28
16–30 June	9.89	14.54	26.17	32.43	15.46
1–15 July	20.88	31.58	57.94	51.80	48.45
16–31 July	49.45	48.62	70.09	66.22	50.52
1–15 August	43.96	48.12	63.55	62.61	29.90
16–31 August	14.29	27.32	65.42	59.46	69.07
1–15 September	0.00	0.75	17.76	12.61	63.92
Don’t remember	29.67	19.80	14.95	15.32	4.12
Respondents reporting heat (N)	91	399	107	222	97

When asked to describe the most recent heat spell experienced in their area, only 8.53% agreed that it was “very hot, but dry.” The most frequently selected category was “very hot and humid” (83.68%), 13.66% chose “the temperature was not excessively high, but it was extremely humid”, about 18% bemoaned the lack of a breeze, and 23.52% said that “the night was too warm”. About one-third of the respondents considered this episode “unusual for this time of the year”. The share of respondents who regard this heat spell as unusual varies across location, ranging from 17.11% in the Regina, SK, area to 46.90% in Sarnia, ON, and 70.19% in Fredericton, NB—the normally cool city with an unusually hot early September. 

How did our respondents cope with this particular heat spell? About 58% spent most of the time in an air-conditioned environment, 31% stayed inside, almost 50% stayed hydrated, 9% spent most of the time at a swimming pool or another body of water, and 11% spent most of the time in the shade. Some respondents had to be outside because of their job (7.6%), and 13.76% said that they did nothing in particular because they enjoy the heat or are not sensitive to it.

## 6. Heat-Related Illness

Did this particular heat episode affect our respondents’ health? Twenty percent of the respondents said it did. We further asked these respondents (and those who were not sure if they had been sick because of the heat) to describe their symptoms. The responses provided by these 243 respondents are summarized in Table 7, along with the median age of the respondents experiencing such symptoms.

**Table 7:** Symptoms reported by respondents whose health was affected by the most recent heat spell (N = 243).

	N	Percent of the sample	Median age of the respondents reporting the symptoms
felt dizzy	91	37.45	44
felt nauseous	98	40.33	48
difficulty breathing	94	38.68	51
became dehydrated	65	26.75	43
mild heat exhaustion	84	34.57	45
severe heat exhaustion	9	3.70	53
heat stroke	8	3.29	42
other cardio- or cerebro-vascular symptoms	14	5.76	49
other illnesses or symptoms	58	23.87	49

We note that out of the 84 people who reported a “mild heat exhaustion,” only in 4 cases was such a condition diagnosed by a health care professional. Of the 9 reported “severe heat exhaustion” cases, only 3 were confirmed by a health care professional. The ages of these persons ranged from 51 to 68, and the average was almost 58. Of the 14 cases of cardiovascular or cerebrovascular symptoms, 7 were diagnosed by a health care professional. Fourteen people had “other” illnesses or symptoms diagnosed by a health care professional and 44 people went undiagnosed.

In sum, 25 people had their illnesses diagnosed by a health care professional. Eleven (44%) regarded these illnesses as minor, in the sense that they really did not change their daily routine. Nine (36%) considered them significant, because they had to make changes to daily routine, and for five people (20%) they were major, because they had to go to the hospital, health clinic or similar facility. 

We checked whether the incidence of illness during the most recent heat spell varied across the study areas, and indeed it did. As shown in Table 8, in the Ontario cities about 28% of the respondents were physically ill during the most recent heat episode. By contrast, barely 10% of the Regina, SK, respondents had been sick. Just over 27% of the Winnipeg residents and 21% of the Fredericton residents experienced illness during the most recent heat episode. 

**Table 8:** Illness during the most recent heat spell by area of residence.

	Regina, SK	Winnipeg, MB	Sarnia, ON	Windsor, ON	Fredericton, NB	Row total
Yes	9.87	17.76	27.43	29.26	21.15	20.49
No	87.5	79.17	69.91	69.43	75.96	76.94
Don’t know	2.63	3.07	2.65	1.31	2.88	2.56
Column Total (N)	152	456	113	229	104	1054

We had expected the illness rate during the latest heat episode to be highest among the elderly, but this expectation is not borne out in the data. Table 9 shows that persons in the 25–34 and 35–54 age group actually reported higher rates of illness during the most recent heat wave than 55-year-olds and older (around 15–16%). Perhaps younger persons spent more time outdoors during the hot weather days, confused bad air quality and the associated symptoms with heat-related symptoms, and/or simply misclassified their illnesses.

**Table 9:** Illness experienced during the most recent heat spell by age group.

	25 to 34	35 to 54	55 to 64	65 to 74	75+	Row total
Yes	21.76	23.52	16.48	16.34	15	20.49
No	74.61	73.94	81.25	81.05	85	76.94
Don’t know	3.63	2.54	2.27	2.61	0	2.56
Column Total (N)	193	472	176	153	60	1054

Epidemiologic studies have linked heat-related illnesses to poor cardiovascular and respiratory health status, and so we are not surprised to see that, as shown in Table 10, people with compromised cardiovascular and respiratory health reported a higher incidence of heat-related illnesses that people who do not have chronic conditions. 

**Table 10:** Heat-related illnesses and health status of the respondents.

Description	Number of persons	Percentage reporting symptoms during most recent heat spell
Diabetes	119	24.37
high blood pressure	312	21.47
high LDL cholesterol	290	23.1
Coronary	46	41.3
Angina	39	46.15
myocardial infarction (heart attack)	34	38.24
Stroke	22	31.82
any other cardiovascular illness	28	42.86
Emphysema	12	50.00
chronic bronchitis	47	42.55
Asthma	143	30.07
other respiratory conditions	76	44.74
Cancer	59	22.03
none of the above illnesses	513	14.23

Out of the 188 people who said that they experienced this illness in 2010 (47.72% of persons who ever experienced a heat-related illness), 162 indicated that they got sick in July or August. A total of 6 people said that they experienced this illness in January through May 2010—presumably during the early heat wave at the end of May—and 12 people in September.

## 7. The Mitigating Effect of Air Conditioning

Air conditioners and other cooling devices have a strong protective effect during heat waves. This section of the paper reports about the rate of penetration of home air conditioning and examines the association between air conditioning and heat-related illness. 

Table 11 shows that almost two-thirds of the sample has a central air conditioning (A/C) system at home, and almost 22% has window units. Only about 14 percent of the sample has neither. 

**Table 11:** Air conditioning at home.

	N	Percent
central A/C	740	64.86
Window-unit A/C	248	21.74
no A/C	162	14.20

Table 12 shows that, as one might expect, there is considerable variation across locales in the prevalence of and type of A/C at home. In Windsor, ON, 84% of the respondents have central A/C, 16% have window units, and 3% have neither. But in Fredericton, NB, central A/C is available only to 11% of the respondents, 41% has window units, and 47% has no A/C at all. At the remaining locations, the central A/C rate ranges from 60 to 69%, window units range from 13% to 29%, and the share of homes without A/C ranges from 10% to 24%.

**Table 12:** Air conditioning by area of residence.

	Regina, SK	Winnipeg, MB	Sarnia, ON	Windsor, ON	Fredericton, NB	Row Total (N)
central A/C	62.43	69.46	60.68	84.03	11.61	740
window-unit A/C	13.29	21.36	29.06	15.97	41.07	248
No	24.28	9.38	10.26	3.36	47.32	162
Column Total (N)	173	501	117	238	112	1141

Of the other cooling devices, fans are common (87% of the sample), but only 6 respondents have a swamp cooler. Out of the 210 respondents who live in housing other than single-family homes or semi-attached homes, 78 said that there is a lobby or common area with A/C, 118 did not have such common area with A/C, and 14 did not know. 

Table 13 indicates that there is no particular association between the presence of air conditioning in one’s home and heat-related illness: The share of respondents who experienced illness is virtually the same across the groups with and without air conditioning at home. (A chi square statistic of 0.0209, p value 0.885, fails to reject the null of independence between illness and air conditioning.)

**Table 13:** Heat-related illness and availability of air conditioning at home.

AC at Home	Has had heat-related illness during latest heat spell (row percentage)		Total
	no	Yes	
No	793 (81.00)	186 (19.00)	979 (100.00)
Yes	132 (81.48)	30 (18.52)	162 (100.00)
Total	925	216	1141

Table 14 reports two probit regressions where the dependent variable is having had a heat-related illness during the most recent spell. Model (A) shows no evidence of an association with the presence of air conditioning at home. Model (B) enters dummies for the city of residence of the respondent, gender, age group, and the fact that the respondent has one or more of the chronic illnesses listed in Table 2. The presence of air conditioning at home remains insignificant, but all of the other variables are significantly associated with having being ill. Specifically, the residents of the other four cities are more likely, all else the same, to report a heat-related illness than the residents of Regina. The effect is especially strong among Windsor residents. Men are less likely to report heat-related illnesses, and persons in all age groups are less likely to have had heat-related illnesses than the 30-year-olds and younger respondents. This confirms the results in Table 13, and suggests that precautions and behaviors might be behind these findings. Persons with chronic illnesses are, all else the same, more likely to experience symptoms during extremely hot periods. 

**Table 14:** Probit regressions. Dep. Var. = Having had a heat-related illness during the most recent heat spell (dummy).

Sick	(A)		(B)	
	coeff.	t stat	coeff.	t stat
Intercept	−0.87793	−19	−1.10241	−5.36
No AC at home	−0.01785	−0.14	0.026307	0.19
Winnipeg			0.430236	2.69
Sarnia			0.74479	3.86
Windsor			0.80003	4.68
Fredericton			0.557181	2.76
Male			−0.41312	−4.42
age3039			−0.38465	−2.22
age4049			−0.30068	−1.8
age5059			−0.31674	−1.91
age6069			−0.6049	-3.3
age70plus			−0.81315	−3.76
Chronic illness			0.446426	4.53
log L	−553.623		−511.179	

## 8. Warnings and Coping with Excessive Heat

Our survey data suggest that when the most recent heat spells occurred, most people (87.38%) were aware that it was going to be extremely hot ahead of time. They had heard it on the weather forecast (905 out of these 921 persons, or 98.26%) [37], or from family and friends (104 out of 921, or 11.29%). A smaller number of respondents said that they had heard it from a Canadian government outreach and information program (16 people, or 1.74%) or from the city (15 people, or 1.63%), and 27 people said that they heard it in other ways (2.93%). 

We asked respondents what they would do if they heard that, starting the next day, there would be a three-day heat wave. As shown in Table 15, respondents propose to stay inside and use the A/C, or stay inside, avoid strenuous activities and drink plenty of water. These measures are even more common with one’s children or any elderly person that the respondent is responsible for.

**Table 15:** Coping with a heat spell starting tomorrow. Percentage of the respondents who would…

Option	For oneself	For one’s children under 12	For the elderly in care of the respondent
stay inside and use a fan	15.43	13.45	24.27
stay inside and use the A/C	53.29	68.16	78.16
stay inside, drink water, avoid strenuous activities	39.18	45.29	48.06
go to a pool or body of water	14.29	52.91	2.91
go to the movies	3.86	9.87	1.94
go to a shopping mall	8.50	13.45	6.80
get out of town to a cooler area	3.51	5.83	1.94
Other	4.91	4.93	1.46
nothing in particular	20.33	4.93	3.88
Number of respondents	1141	223	206

Taking the children to a pool or a body of water was a popular option for parents (53%), but was selected much less frequently for oneself (14%) or for one’s elderly charges (2.9%). Movies and shopping (both air-conditioned places) were not popular selections for the respondents or the elderly, but 13% of the parents indicated that they might take (or send) their children to a shopping mall. The option of going out of town was selected by relatively few respondents. In general, respondents tended to be somewhat more proactive with their children and any elderly persons they take care of than for themselves.

The respondents seemed realistic in their assessment of what they would do to protect themselves from excessive heat. As shown in Table 16, those who have air conditioning at home are much more likely to stay inside and use air conditioning that those who do not. Those who do not have air conditioning at home would resort to staying inside and using a fan. The respondents’ likelihood of engaging in other activities to protect themselves from the heat was similar across the two groups. Overall, however, those without air conditioning at home were somewhat more likely to indicate that they would do “nothing in particular”.

**Table 16:** Coping with a heat spell starting tomorrow by availability of air conditioning.

Option	For oneself, and has A/C at home	For oneself, and has no A/C at home
stay inside and use a fan	10.11	47.53
stay inside and use the A/C	61.39	4.32
stay inside, drink water, avoid strenuous activities	38.30	44.44
go to a pool or body of water	14.50	12.96
go to the movies	3.58	5.56
go to a shopping mall	8.48	8.64
get out of town to a cooler area	3.47	3.70
Other	4.80	5.56
nothing in particular	19.41	25.93
Number of respondents	979	162

## 9. Public Programs and Populations at Risk

Regarding “cooling centers,” 14.20% of our respondents had heard of them in their city, 29.27% had heard of cooling centers in other cities, and almost 59% had never heard of them at all. Only 12 respondents (or 1% of the sample) said that they had used a cooling center before. Three of these respondents live in Regina, 3 in Winnipeg, 1 in Sarnia, 3 in Windsor and 2 in Fredericton.

Table 17 shows that familiarity with cooling center does vary across locales. For example, in the Ontario cities about one-third of respondents have heard of cooling center here and even larger shares of samples have heard of cooling centers at other locations.

**Table 17:** Familiarity with cooling centers by area of residence. Percentage of respondent by city who…

	Regina, SK	Winnipeg, MB	Sarnia, ON	Windsor, ON	Fredericton, NB	Row total (N)
Have heard of cooling centers in own city	3.47	5.59	34.19	31.51	11.61	162
Have heard of cooling centers in other areas	26.59	26.75	40.17	32.35	26.79	334
Haven’t heard of cooling centers	71.1	68.46	32.48	40.76	63.39	672
Column Total (N)	173	501	117	238	112	1141

In Winnipeg, 68% of the sample had never heard of cooling centers. In Regina, the share of the sample who does not know cooling centers is 71%. 

**Figure 2:**
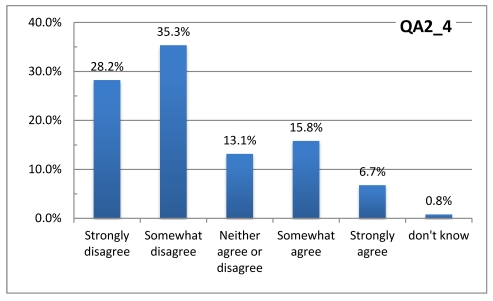
Respondent agreement or disagreement with the statement: “Only people in very poor health are at risk of illness or even death during heat waves”.

**Figure 3:**
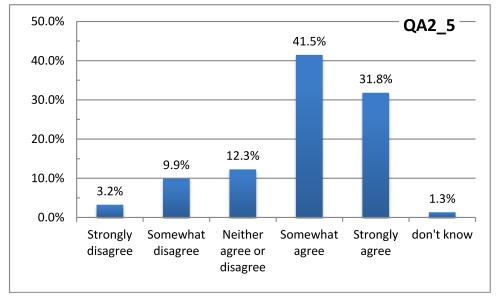
Respondent agreement or disagreement with the statement: “Everyone is at risk of illness or even death during heat waves”.

The questionnaire also inquired about the respondents’ perceptions about individuals and populations at risk during heat waves. Figure 2 and Figure 3 suggest that most of the respondents take the heat waves seriously and believe that the population at large is at risk—not just persons in poor health. This evidence is in sharp contrast with Sheridan [18] where many subjects did not consider themselves at risk.

## 10. Conclusions

We have conducted a survey of households in five cities in Canada to assess their perceptions of excessive summer heat, experience with heat-related illnesses, and coping mechanisms. The survey was conducted in the summer of 2010. At the time of the survey the cities had not yet implemented formal heat illness outreach program or heat response programs. 

Our respondents were generally well aware of the heat spells in that summer, and 21% of them reported having been unwell because of the heat. The majority of these episodes of heat-related illness, however, were relatively minor. Our survey confirms the medical and epidemiological literature, in that the respondents who report the highest rates of heat-related illness during the most recent heat wave are those in compromised cardiovascular and respiratory health. Surprisingly, younger respondents were *more* likely to report heat-related illness, and air conditioning bore no relationship with the likelihood of experiencing a heat-related illness, whether or not we controlled for the area of residence of the respondent, gender, and existing chronic illness conditions. Since the protective role of air conditioning is well-established, we suspect that result may be due to confusion about the true nature and cause of the illness (heat waves are often accompanied by severe air pollution episodes), misclassification of symptoms, or simply spending time outdoors. Indeed, our probit regressions suggest that, even controlling for respondent characteristics and air conditioning at home, illness rates are somewhat higher at locales where, for any given temperature, the heat index and/or humidex would be higher (e.g., in Ontario).

When asked how they would protect themselves, their young children and any elderly person that they are in charge of from a hypothetical heat wave that starts tomorrow, our respondents appear to be well aware of common-sense, medically recommended protections, such as staying in climate-controlled environments, keeping hydrated, *etc.*—even though a formal outreach program had not been initiated yet. Most likely the media and other sources have provided plentiful information about heat mitigation. Our respondents seemed especially proactive in seeking such protections for their young children and any elderly they are responsible for. In general, they tended to think that virtually everyone is at risk during heat waves, not just persons in compromised health.

Our survey also demonstrated that air conditioning is not ubiquitous in Canada: in the Fredericton area, about 47% of our respondents did not have air conditioning in their home, a figure that is in sharp contrast with those in our Ontario cities and Winnipeg. Our respondents were also relatively unaware of “cooling centres,” since 59% had never heard of them and only 1% had used them before. These results and the other findings of this study suggest that outreach initiatives that inform individuals about cooling centers and similar options, and emphasize the importance of limiting exposure to hot and humid conditions, will be especially useful in reducing heat-related illness.

## Supplementary Files

Supplementary File 1: DOCX-Document (DOCX, 33 KB)
