# Supplementary material for: Individual and Public-Program Adaptation: Coping with Heat Waves in Five Cities in Canada
Source: Int J Environ Res Public Health. 2011 Dec 16;8(12):4679–701. doi: 10.3390/ijerph8124679 (PMC3290981; doi:10.3390/ijerph8124679)
Supplement: Supplementary File 1: — DOCX-Document (DOCX, 33 KB) [file ijerph-08-04679-s001.docx]

**Appendix. Survey Questionnaire**

Heat Effects Baseline Survey: Summer 2010

—Final Questionnaire—

August 25, 2010

**[Introduction at Site]**

Thank you for your participation in our Ipsos i-Say online surveys. Your opinions are very important to us.

This survey will take you about 15 minutes and you’ll earn up to 90 reward points upon completing it.

You’ll also get the chance to play our Poll Predictor game, with prizes awarded each week. And that’s not all! Each time you click on a survey link you will receive an entry into our monthly contest awarding $5000. Click here for full contest rules and regulations.

Please remember, all information you provide is kept strictly confidential and is used for research purposes only.

**[Screening]**

**S1.** What is your gender?

Male

Female

**[TRACK GENDER QUOTAS BASED ON S1]**

**S2.** What is your date of birth (year and month)?

Year: [DROP-DOWN BOX [RANGE: 1910 TO 2009]

Month: [DROP-DOWN BOX. RANGE: January to December]

**[THANK & TERMINATE IF 24 OR YOUNGER. TRACK AGE QUOTAS BASED ON S2]**

**S3.** In which of the following cities (**or surrounding areas**) do you usually reside?

*Please select one response only*

Regina, Saskatchewan

Winnipeg, Manitoba

Sarnia, Ontario

Windsor, Ontario

Fredericton, New Brunswick

None of the above

**[THANK & TERMINATE IF NONE OF THE ABOVE. TRACK CITY QUOTAS BASED ON S3.]**

**[Section 1: Experience with Excessive Heat]**

We will be asking you a few questions about what you did and how you felt this past summer. By **summer**, we mean the period from **June 1, 2010 to present**.

**1.** Which of the following best describes where you spent this past summer–that is, June 1 to present?

*Please select one response only*

I spent all summer in the **[INSERT CITY FROM S1:** Regina, Winnipeg, Sarnia, Windsor, Fredericton**]** area

I spent most of the summer in the **[INSERT CITY FROM S1]** area, with occasional out-of-town trips

I spent most of the summer in the **[INSERT CITY FROM S1]** area, but I took a vacation of a week or longer elsewhere

I spent most of the summer away from the **[INSERT CITY FROM S1]** area, such as at a vacation home

Other (Please specify)

**[ASK Q2 IF ‘I SPENT ALL SUMMER OR MOST OF THE SUMMER IN THE CITY FROM S1 AREA’ IN Q1, ELSE SKIP TO Q4]**

**2.** When you were in the **[INSERT CITY FROM S1]** area this past summer, were there any times when the weather felt unbearably hot and oppressive to you?

*Please select one response only*

Yes

No

**[ASK Q3A & Q3B IF YES IN Q2, ELSE SKIP TO Q4]**

**3A.** Again thinking about the time you spent in the **[INSERT CITY FROM S1]** area, about how many episodes of unbearably hot and oppressive weather do you recall this past summer? By ‘episode,’ we mean one or more consecutive days of oppressively hot weather.

*Please provide your best estimate*

NUMERIC RESPONSE [RANGE: 1 TO 100]

**3B.** Do you remember the approximate date(s) when this happened?

*Please select all that apply*

June 1 to 15

June 16 to 30

July 1 to 15

July 16 to 31

August 1 to 15

August 16 to 30

September 1 to 15

I do not remember

**4.** Were there any times in **previous** summers (2009 and earlier) when the weather felt unbearably hot and oppressive to you in the **[INSERT CITY FROM S1]** area?

*Please select one response only*

Yes

No

I did not live in the **[INSERT CITY FROM S1]** area in 2009 or earlier

**[IF (I SPENT MOST OF THE SUMMER AWAY FROM THE CITY FROM S1 AREA AT Q1 OR OTHER AT Q1 OR NO AT Q2) AND (NO OR I DID NOT LIVE IN… AT Q4), SKIP TO Q14]**

**[DISPLAY Q5A AND Q5B ON THE SAME SCREEN]**

**5A.** Thinking about the **most recent** heat spell you experienced in the **[INSERT CITY FROM S1]** area, approximately when did that occur?

*Please select one response only*

June 1 to 15, 2010

June 16 to 30, 2010

July 1 to 15, 2010

July 16 to 31, 2010

August 1 to 15, 2010

August 16 to 30, 2010

September 1 to 15, 2010

In 2009

In 2008

In 2007 or earlier

I do not remember

**5B.** And approximately how many days did the heat spell last?

*Please provide your best estimate*

NUMERIC RESPONSE [RANGE: 1 TO 100]

I do not remember

**6.** Still thinking about the **most recent** heat spell you experienced in the **[INSERT CITY FROM S1]** area, how would you describe it?

*Please select all that apply*

It was very hot, but dry

It was very hot and humid

The temperature was not excessively high, but it was extremely humid

There was no breeze

The night was too warm

Other (Please specify)

**7.** In your experience, would you say that the heat spell was unusual for the **[INSERT CITY FROM S1]** area at that time of the year?

*Please select one response only*

Yes

No

Don’t know

**8.** Still thinking about the **most recent** heat spell you experienced in the **[INSERT CITY FROM S1]** area, did you experience physical discomfort or illness as a result of this episode?

*Please select one response only*

Yes

No

**[ASK Q9 IF YES AT Q8, ELSE SKIP TO Q12]**

**9.** How severe was the physical discomfort that you experienced during this most recent heat spell?

*Please select all that apply*

I did not experience any actual discomfort because I was able to spend most of the time in an air conditioned environment

I did not experience any actual discomfort because I was able to stay hydrated

I did not experience any actual discomfort (other)

I was uncomfortable because of the heat and/or humidity, but I did not experience any illness

I became dehydrated

I had mild heat exhaustion

I had severe heat exhaustion

I had heat stroke

I had cardiovascular or cerebrovascular symptoms

I had other symptoms/got sick (Please specify)

**[ASK Q10A IF MILD HEAT EXHAUSTION SELECTED IN Q9. ASK Q10B IF SEVERE HEAT EXHAUSTION SELECTED IN Q9. ASK Q10C IF CARDIOVASCULAR OR CEREBROVASCULAR SYMPTOMS SELECTED IN Q9. ASK Q10D IF OTHER SYMPTOMS/GOT SICK SELECTED IN Q9] [SHOW ALL Q10 ITERATIONS ON THE SAME SCREEN]**

**10A.** Was your mild heat exhaustion diagnosed by a health care professional?

*Please select one response only*

Yes

No

**10B.** Was your severe heat exhaustion diagnosed by a health care professional?

*Please select one response only*

Yes

No

**10C.** Were your cardiovascular or cerebrovascular symptoms diagnosed by a health care professional?

*Please select one response only*

Yes

No

**10D.** Were your other symptoms/illness diagnosed by a health care professional?

*Please select one response only*

Yes

No

**[ASK Q11 IF YES IN Q10A, Q10B, Q10C OR Q10D, ELSE SKIP TO Q12]**

**11.** How severe were these symptoms/ this illness that was diagnosed by a health care professional?

*Please select one response only*

Minor – it didn’t really change my daily routine

Significant – I couldn’t carry out my daily routines (e.g., couldn’t go to work, *etc*.)

Major – I had to go to the hospital, health clinic or similar health centre

**12.** During the **most recent** heat spell you experienced in the **[INSERT CITY FROM S1]** area, did you know ahead of time that it was going to be very hot for one or more days?

*Please select one response only*

Yes

No

I do not remember

**[ASK Q13 IF YES IN Q12, ELSE SKIP TO Q14]**

**13.** How did you find out that it was going to be very hot for one or more days?

*Please select all that apply*

Weather forecast

I heard it from friends or others

Canadian government outreach and information program

City outreach and information program

Other (Please specify)

**14.** How frequently did you check the weather forecast this past summer?

*Please select one response only*

Every day, several times a day

Every day, once a day

Once every few days

Once a week

One to three times a month

Less than once a month

Never

**15.** How do you normally get the weather forecast?

*Please select all that apply*

TV

Radio

Newspapers

SMS or RSS feeds

Internet

Through friends and family

Other

I do not check the weather forecast

**16.** What would you do if you heard that it was going to be extremely hot and uncomfortable for 3 consecutive days in the **[INSERT CITY FROM S1]** area, starting the next day?

*Please select all that apply*

I would make sure to stay inside as much as possible and use a fan

I would make sure to stay inside as much as possible and use the air conditioner (A/C)

I would make sure to stay inside as much as possible, drink plenty of water and avoid strenuous activities

I would try to spend as much time as possible near or in a swimming pool or other body of water

I would go to the movies because movie theaters are usually air conditioned

I would go shopping because shopping malls are usually air conditioned

I would try to get out of town and go to a cooler area

Other (Please specify)

I would do nothing in particular

**17A.** Do any children under the age of 12 live in your home on a regular basis?

*Please select one response only*

Yes

No

**[ASK Q17B IF YES TO Q17A, ELSE SKIP TO Q18A]**

**17B.** Thinking about the children in your home under the age of 12, what would you do to keep them cool and healthy if you heard that it was going to be extremely hot and uncomfortable for 3 consecutive days in the **[INSERT CITY FROM S1]** area, starting the next day?

*Please select all that apply*

I would keep them inside as much as possible and use a fan

I would keep them inside as much as possible and use the air conditioner (A/C)

I would keep them inside as much as possible, provide plenty of water to drink and avoid strenuous activities

I would take them to a swimming pool or other body of water

I would take them to the movies because movie theaters are usually air conditioned

I would take them shopping because shopping malls are usually air conditioned

I would try to get out of town and go to a cooler area

Other (Please specify)

I would do nothing in particular

**18A.** Do you have elderly parents, relatives or other elderly adults that you take care of who live in the **[INSERT CITY FROM S1]** area?

*Please select one response only*

Yes

No

**[ASK Q18B IF YES IN Q18A, ELSE SKIP TO Q19A]**

**18B.** Thinking about the elderly individuals you take care of, what would you do to keep them cool and healthy if you heard that it was going to be extremely hot and uncomfortable for 3 consecutive days in the **[INSERT CITY FROM S1]** area, starting the next day?

*Please select all that apply*

I would keep them inside as much as possible and use a fan

I would keep them inside as much as possible and use the air conditioner (A/C)

I would keep them inside as much as possible, provide plenty of water to drink and avoid strenuous activities

I would take them to a swimming pool or other body of water

I would take them to the movies because movie theaters are usually air conditioned

I would take them shopping because shopping malls are usually air conditioned

I would try to get out of town and go to a cooler area

Other (Please specify)

I would do nothing in particular

**19A**. In some cities where most homes do not have air conditioning, the city government provides “cooling centres” – that is, public buildings with air conditioning where people can go and spend time in an air conditioned environment during excessive heat spells. Have you ever heard of cooling centres in **[INSERT CITY FROM S1]** or in any other city or town?

*Please select all that apply*

Yes – I have heard of cooling centres in the **[INSERT CITY FROM S1]** area

Yes – I have heard of cooling centres in another city or town

No

**[ASK Q19B IF YES IN Q19A ELSE SKIP TO INSTRUCTION BEFORE Q20]**

**19B.** Have you ever used a cooling centre?

*Please select one response only*

Yes

No

**[IF YES – I HAVE HEARD OF COOLING CENTRES IN CITY FROM S1 AT Q19A, SKIP TO Q21, ELSE CONTINUE WITH Q20]**

**20.** How do you think you would find out if the **[INSERT CITY FROM S1]** area has a cooling centre?

*Please be specific and detailed in your response*

[VERBATIM RESPONSE]

**21.** Have you, or anyone you know, ever felt sick during an excessive heat spell?

*Please select all that apply*

Yes – I have felt sick

Yes – A family member or someone else I know has felt sick

No

**[ASK Q22A, Q22B & Q22C IF YES – I HAVE FELT SICK IN Q21, ELSE SKIP TO Q23. DISPLAY Q22A, Q22B & Q22C ON THE SAME SCREEN]**

**22A.** Thinking about the last time you felt sick from excessive heat, how severe was the illness?

*Please select one response only*

Minor – it didn’t really change my daily routine

Significant – I couldn’t carry out my daily routines (e.g., couldn’t go to work, *etc*.)

Major – I had to go to the hospital, health clinic or similar health centre.

**22B.** Approximately how long did this illness last?

*Please select one response only*

Less than one day

One day

Two or three days

Four to six days

One week

Two weeks

Three weeks

Four weeks

Longer than four weeks

I don’t remember

**22C.** Approximately when did this happen?

Month [DROP-DOWN BOX. RANGE: JANUARY TO DECEMBER]

Year [DROP-DOWN BOX: 2010, 2009, 2008, 2007, 2006, 2005 or earlier]

I don’t remember

**23.** Excessive heat can make people sick. Where would you look for information about the possible illnesses caused by extremely hot weather?

*Please select all that apply*

[RANDOMIZE ORDER]

Family doctor

Nurse

Public health clinic

Pharmacist

Books or journals

Magazines

Internet

Library

Family/friends/colleagues

Other (please specify)

Don’t know

**[Section 2: Attitudes]**

**A1.** The following is a list of some issues facing Canadians. For each issue, would you say Canadians should be doing a lot less, somewhat less, about the same, somewhat more or a lot more than we are today?

*Please select one response for each item*

[DOWN SIDE OF GRID. RANDOMIZE ORDER.] **[PROGRAMMER NOTE: PLEASE INCLUDE A VARIABLE TO KEEP TRACK OF THE ORDER OF PRESENTATION OF EACH ITEM FOR EACH RESPONDENT]**

Reduce air and water pollution

Maintain parks and wildlife reserves

Protect species at risk

Improve roads and highways

Encourage economic growth and jobs

Improve health care and prevention

Improve education

Address climate change

Reduce taxes

[ACROSS TOP OF GRID]

Do a lot less

Do less

Do about the same

Do more

Do a lot more

Not sure

**A2.** Please indicate your agreement or disagreement with each of the following statements.

*Please select one response for each item*

[DOWN SIDE OF GRID. RANDOMIZE ORDER.] **[PROGRAMMER NOTE: PLEASE INCLUDE A VARIABLE TO KEEP TRACK OF THE ORDER OF PRESENTATION OF EACH ITEM FOR EACH RESPONDENT]**

During extreme weather events, people should be responsible for themselves

It is the government’s responsibility to protect people from extreme weather events

Government intervention can be effective in improving health and safety

Only people in very poor health are at risk of illness or even death during heat waves

Everyone is at risk of illness or even death during heat waves

[ACROSS TOP OF GRID]

Strongly disagree

Somewhat disagree

Neither agree nor disagree

Somewhat agree

Strongly agree

Not sure

**A3.** Below is a list of **possible threats to the health and safety of Canadians**. For each, please indicate how much of **a threat or risk** you feel each of these is to you personally.

*Please select one response for each item*

[DOWN SIDE OF GRID. RANDOMIZE ORDER.] **[PROGRAMMER NOTE: PLEASE INCLUDE A VARIABLE TO KEEP TRACK OF THE ORDER OF PRESENTATION OF EACH ITEM FOR EACH RESPONDENT]**

Forest fires

Air pollution

Traffic accidents

Weather extremes (storms, wind)

Natural disasters (earthquakes, *etc*.)

Extreme heat

Terrorism

Workplace accidents

[ACROSS TOP OF GRID]

Very low risk

Low risk

Moderate risk

High risk

Very high risk

Don’t know

**[Section 3: Housing and Neighbourhood]**

The final few questions are for statistical calculations. Please be assured all information will be kept completely confidential.

**RH1.** Which of the following best describes the area where you live?

*Please select one response only*

An acreage, ranch or farm

A town

Suburbs of a city

In a city

**RH2.** Which of the following best describes your principal home?

*Please select one response only*

Single-family home

Duplex, triplex, fourplex or townhouse (semidetached or attached home)

Apartment/condominium in a residential building

Seniors’ apartment or residence without assisted living care services

A nursing home or other type of assisted living

Other (Please specify)

**RH3.** If you live in a multi-floor building, what floor do you live on?

*Please select one response only*

DROP-DOWN BOX [RANGE: 1 to 50]

I don’t live in a multi-floor building [DISPLAY BELOW DROP-DOWN BOX]

**RH4.** Does your home have air conditioning?

*Please select all that apply*

Yes, central system

Yes, window unit

No

**RH5.** Does your home have a swamp cooler?

*Please select one response only*

Yes

No

Don’t know

**RH6.** Do you have fans at home (electric fans or ceiling fans)?

*Please select one response only*

Yes

No

**RH7.** If you live in an apartment building, a high-rise, another type of multi-family residential building, or a nursing home, is there a lobby, hallway, or another room accessible by the residents that has air conditioning?

*Please select one response only*

Yes

No

Don’t know

I don’t live in these types of dwellings

**RH8.** Do you have…?

*Please select all that apply*

A shaded backyard or garden

A lawn

Trees in your backyard or front yard

Trees in your neighborhood, in the immediate vicinity of your home

None of the above

**[Section 4: Personal Health]**

**D1.** Compared to other people your age, would you say that your health is...?

*Please select one response only*

Excellent

Very good

Good

Fair

Poor

**D2.** Has a doctor ever told you that you have any of the following...?

*Please select one response for each item*

[DOWN SIDE OF GRID – DO NOT RANDOMIZE]

Diabetes

High blood pressure, also known as hypertension

High levels of LDL cholesterol (the so-called “bad” cholesterol)

Coronary disease

Angina (also known as angina pectoris)

A myocardial infarction/heart attack

A stroke

Any other cardiovascular disease

Emphysema

Chronic bronchitis

Asthma

Other respiratory problems

A malignant tumor (cancer) of any type

[ACROSS TOP OF GRID]

Yes

No

Prefer not to say

**[ASK D3 IF YES TO ANY ITEM IN D2, ELSE SKIP TO D4]**

**D3.** Are you being treated or about to be treated for this illness/these illnesses?

*Please select one response only*

Yes

No

Prefer not to say

**D4.** In the last 5 years, have you been admitted to the hospital or taken to the emergency room because of cardiovascular or respiratory problems, or cancer?

*Please select one response only*

Yes

No

Don’t know

**[ASK D5 AND D6 IF YES IN Q18A, ELSE SKIP TO D7] [SHOW D5 AND D6 ON THE SAME SCREEN]**

**D5.** Earlier you indicated that you have elderly parents, relatives or other elderly adults that you take care of. Do any of the elderly persons have a chronic cardiovascular or respiratory condition, such as heart disease, chronic bronchitis, or emphysema?

Yes

No

**D6.** Do any of these elderly persons have an impairment that prevents them from getting around and taking care of themselves?

Yes—minor

Yes—moderate

Yes—major

No

**D7.** Do any other members of your household (excluding yourself) have a chronic cardiovascular or respiratory condition, such as heart disease, chronic bronchitis, or emphysema?

Yes

No

**D8.** At the present time, do you smoke cigarettes daily, occasionally, or not at all?

*Please select one response only*

Daily

Occasionally

Not at all

Prefer not to say

**[SKIP TO D9B IF DAILY OR OCCASIONALLY SELECTED IN D8, ELSE CONTINUE WITH D9A]**

**D9A.** Have you ever smoked cigarettes daily or occasionally?

*Please select one response only*

Yes – daily

Yes – occasionally

No

Prefer not to say

**[ASK D9B IF DAILY OR OCCASIONALLY IN D8. ASK D9C IF YES DAILY OR YES OCCASIONALLY IN D9A. ALL OTHERS SKIP TO D10.]**

**D9B.** For how long have you smoked cigarettes??

*Please select one response only*

Less than one year

One to two years

Three to five years

Six to ten years

More than ten years

**D9C.** When did you stop smoking cigarettes?

*Please select one response only*

Less than one year ago

One to two years ago

Three to five years ago OR

More than five years ago

**D10.** Do any other members of your household smoke cigarettes daily or occasionally?

Yes – daily

Yes – occasionally

No

**D11.** On average, approximately how much time do you devote to acquiring health information? This includes information from television, newspapers, magazines, websites, books, journals, doctors, dietitians or other health professionals, libraries, non-profit organizations, government departments, or other sources.

*Please select one response only*

More than 5 hours per week

2 to 4 hours per week

1 hour per week

At least 1 hour per month

Less than 1 hour per month

Don’t know

**D12.** Do you receive any of the following services (provided by the government or a voluntary organization)?

*Please select all that apply*

Meals-on-Wheels

Home delivery of groceries

Home delivery of medicines

Regular doctor visits at home

I live in a nursing home or other type of assisted living, and so I receive some or all of these services as part of living there

None of the above

**[Section E: You and Your Household]**

**E1.** DELETED

**E2.** DELETED

**E3.** Which of the following best describes your marital status?

*Please select one response only*

Married/cohabitating with partner

Separated or divorced

Widowed

Single (never been married)

Prefer not to say

**E4.** What is the highest level of education that you have completed?

*Please select one response only*

Less than high school

Some high school

Completed high school

Some college or post-secondary technical school

Completed college or post-secondary technical school

Some university (not completed)

Completed university undergraduate degree

Some university post graduate or professional school

Completed university post graduate or professional school degree

Prefer not to say

**E5.** Which of the following best describes your employment status?

*Please select all that apply*

Working full-time (35+ hours per week, including self-employed)

Working part-time (less than 35 hours per-week, including self-employed)

Unemployed but looking for work

Full-time student

Part-time student

Homemaker

Retired

Other

Prefer not to say

**E6.** Including yourself, how many **adults** (18 and older) live in your household?

*Please select one response only*

One

Two

Three

Four

Five

Six

Seven

Eight

Nine or more

Prefer not to say

**E7.** How many **children** in each of the following age groups live in your household?

*Please select one response for each item*

Aged 5 or younger [DROP-DOWN BOX: 0, 1, 2, 3, 4, 5, 6, 7, 8, 9 or more, Prefer not to say]

Between the ages of 6 and 12 [DROP-DOWN BOX: 0, 1, 2, 3, 4, 5, 6, 7, 8, 9 or more, Prefer not to say]

Between the ages of 13 and 17 [DROP-DOWN BOX: 0, 1, 2, 3, 4, 5, 6, 7, 8, 9 or more, Prefer not to say]

**[DO NOT MAKE MANDATORY FROM THIS POINT FORWARD]**

**E8.** Which of the following categories best describes the total annual income for all members of your household, before taxes?

*Please select one response only*

Less than $20,000

$20,000–$29,999

$30,000–$39,999

$40,000–$49,999

$50,000–$59,999

$60,000–$79,999

$80,000–$99,999

$100,000–$124,999

$125,000 or more

**E9.** And finally, please provide the first three digits of your postal code.

*Please ensure it is in letter-number-letter format*

[VERBATIM RESPONSE] **[PROGRAMMER NOTE: ENSURE THE CORRECT FORMAT]**

**[Comments]**

If you would like to make any comments about the survey, please type them in the space below.

[VERBATIM RESPONSE]

**You have completed the survey. Thank you very much for participating. We appreciate your help**
